# Marijuana use and short-term outcomes in patients hospitalized for acute myocardial infarction

**DOI:** 10.1371/journal.pone.0199705

**Published:** 2018-07-11

**Authors:** Cecelia P. Johnson-Sasso, Christine Tompkins, David P. Kao, Lori A. Walker

**Affiliations:** Department of Medicine, Division of Cardiology, University of Colorado Anschutz Medical Campus, Aurora, Colorado, United States of America; University of Tampere, FINLAND

## Abstract

Marijuana use is increasing worldwide, and it is ever more likely that patients presenting with acute myocardial infarctions (AMI) will be marijuana users. However, little is known about the impact of marijuana use on short-term outcomes following AMI. Accordingly, we compared in-hospital outcomes of AMI patients with reported marijuana use to those with no reported marijuana use. We hypothesized that marijuana use would be associated with increased risk of adverse outcomes in AMI patients. Hospital records from 8 states between 1994–2013 were screened for patients with a diagnosis of AMI. Clinical profiles and outcomes in patients with reported use of marijuana were compared to patients without reported marijuana use. Short-term outcomes were defined as adverse events that occurred during hospitalization for an admitting diagnosis of AMI. The composite primary outcome included death, intraaortic balloon pump placement, (IABP), mechanical ventilation, cardiac arrest, and shock. In total, 3,854 of 1,273,897 AMI patients reported use of marijuana. The marijuana cohort was younger than (47.2 vs. 57.2, respectively) and had less coronary artery disease than the non-marijuana cohort. In multivariable analysis including age, race and common cardiac risk factors, there was no association between marijuana use and the primary outcome (p = 0.53), but marijuana users were more likely to be placed on mechanical ventilation (OR (odds ratio) 1.19, p = 0.004). Interestingly, marijuana-using patients were significantly less likely to die (OR 0.79, p = 0.016), experience shock (OR 0.74, p = 0.001), or require an IABP (OR 0.80, p = 0.03) post AMI than patients with no reported marijuana use. These results suggest that, contrary to our hypothesis, marijuana use was not associated with increased risk of adverse short-term outcomes following AMI. Furthermore, marijuana use was associated with *decreased* in-hospital mortality post-AMI.

## Introduction

Medicinal and recreational marijuana use is increasing worldwide, partially due to legislative changes in the United States, Europe and South America. The World Health Organization estimates that approximately 2.5% of the world’s population use cannabis, ten times more than cocaine (0.2%) or opiates (0.2%) [1]. Furthermore, cannabis use is increasing more rapidly than use of either cocaine or opiates. In the United States, nearly 55 million adults use marijuana at least yearly [2]. Of those 55 million adults, 35 million consume marijuana monthly and it is estimated that there are 8.5 million *daily* marijuana users [3]. Thirty states and the District of Columbia currently have laws broadly legalizing marijuana (medicinal or recreational) with an additional 12 states considering marijuana legislation in 2018. With the rapidly changing legal landscape surrounding marijuana, the number of people using marijuana is likely to increase.

There are known benefits of marijuana for treating numerous medical conditions such as cancer, glaucoma, HIV/AIDS and posttraumatic stress disorder [4]. However, with the increase in use, there is an alarming increase in reports of adverse cardiovascular events following marijuana exposure [5–7]. Meta-analysis of over 3,500 participants (38% who reported marijuana use) in the CARDIA study showed a positive correlation between marijuana use, hypertension and dyslipidemia, all of which may contribute to coronary artery disease [8]. In addition, a French study recently analyzed 35 reports of “remarkable” cardiovascular complications following marijuana use [9]. During the 5-year study period (2006–2010) 1979 adverse events were reported, 35 (1.8%) of which were cardiovascular related. The cardiovascular events were categorized into cardiac or extra-cardiac (i.e. vascular) events: acute coronary syndrome (ACS) composed 57% of the reports; heart rate disorders 5.7%; cerebral vascular events 8.6%; and peripheral vascular events 28.7%. Unfortunately, over 25% of these cases resulted in death even though the overall mean age of the patients was only 34.5 years. It is important to note, however, that there were an estimated 1.2 million regular marijuana users in France at the time of the study and that the nearly 2000 reported adverse events represents an extremely low complication rate, perhaps due to under-reporting of marijuana use.

These studies strongly suggest an effect of cannabis on cardiovascular health. Importantly, federal data from 2014 revealed that for the first time, middle-aged Americans were slightly *more* likely to use marijuana than their teenage children; data from the Centers for Disease Control and Prevention reported a 10% decline in regular marijuana use by teenagers (12–17 years) from 2002 to 2014, whereas use increased by 8% in those aged 35–44, by a surprising 50% in Americans aged 45–54, and by a stunning 455% among those aged 55 to 64 years old during the same time period. Age is a well-known risk factor for cardiovascular disease [10] and with rising marijuana consumption in older individuals, it is increasingly likely that patients with common cardiovascular conditions such as AMI will be regular marijuana users.

Few studies, however, have examined the impact of marijuana use on outcomes following cardiovascular incidents such as AMI, and they are limited by small sample sizes [11, 12]. One study aimed at quantifying long-term outcomes in marijuana users post-AMI found no association between marijuana consumption and long-term mortality in 2097 post-AMI patients (109 marijuana users) followed up to 18 years [11]. Recently, Desai, et al [13] analyzed large, weighted dataset with nearly 500,000 index admissions and reported that lifetime odds of acute myocardial infarction were increased 3–8% in marijuana users. Desai et al [13] found no increase in in-hospital mortality in the marijuana users following AMI and the authors were able to identify a number of independent predictors of inpatient mortality in the marijuana using group. These findings underscore the need to further assess the effect of marijuana use on the cardiovascular system. Accordingly, the aim of this retrospective study was to quantify short-term outcomes in marijuana-using and non-using patients hospitalized with AMI. Based on the limited published data, we hypothesized that marijuana use would be associated with worse in-hospital outcomes in AMI patients.

## Methods and materials

All data used in this study are publicly available, therefore the requirement for informed consent was waived. Data sources were administrative databases containing all hospital stays for either all or a majority of hospitals in each of 8 states during the study period (1994–2013). After obtaining an exemption from our local Institutional Review Board, de-identified hospital records were obtained from individual state agencies in California, New York, New Jersey, Vermont, New Hampshire, Colorado, Texas, and West Virginia, and all records were screened for patients who were admitted with a diagnosis of AMI as defined in the International Classification of Diseases-9 Clinical Modification [ICD-9 CM] code 410.xx. De-identified data sources are summarized in S1 Table. Demographic data, non-ST elevation MI (NSTEMI) vs. ST elevation MI (STEMI), comorbid conditions, procedures, and outcomes were quantified using dataset documentation and ICD-9-CM codes. Specific ICD-9 CM codes used for each comorbid condition are enumerated in S2 Table.

**Fig 1:**
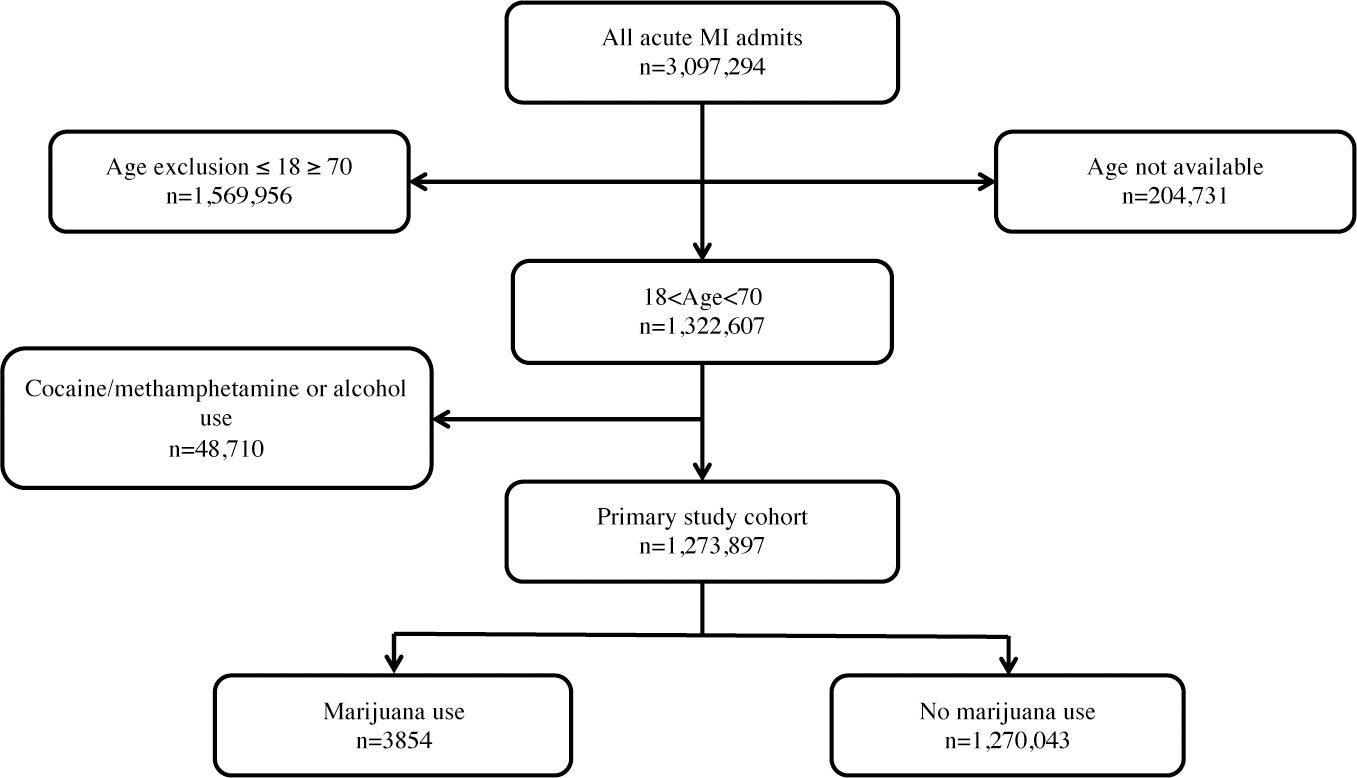
Inclusion algorithm for final study population. Inclusion and exclusion criteria used to select the final study cohort. Individuals ≤ 18 years of age and those ≥ 70 years of age were excluded. Individuals who had used alcohol, cocaine, or methamphetamine prior to admission for acute myocardial infarction were also excluded from the study to reduce confounding co-substance influences.

Over the study period, 3,097,294 inpatient records specified AMI. The characteristics of the original study population are shown in S3 Table. A schematic of the patient inclusion algorithm is shown in Fig 1. Patients ≥ 70 years (n = 1,568,199) were excluded, as a disproportionately large percentage (54%) of control patients were ≥ 70 years compared with marijuana patients (3%). Furthermore, the rate of marijuana use in patients < age 70 (0.5%) was fifty-fold higher than in patients ≥ age 70 (0.01%). Patients ≤ 18 years were excluded (n = 1,757) due to the increased likelihood of congenital heart defects underlying AMI [14]. Patients with reported concomitant use of cocaine/methamphetamine (n = 15,166) or alcohol (n = 68,177) were also excluded from the primary analysis due to possible confounding based on the known cardiotoxic potential of these substances. After these exclusions, 1,273,897 patients admitted with AMI were included in the primary analysis. The primary end-point was a composite of death, mechanical ventilation, cardiac arrest, placement of an intraaortic balloon pump (IABP) or shock. Secondary outcomes included the individual components of the primary outcome, coronary angiogram, coronary percutaneous intervention, and STEMI vs. NSTEMI.

### Statistical analysis

Comparisons of categorical demographic data and comorbidities between the marijuana and control cohort, as well as between patients with and without the primary outcome were performed using Pearson’s chi-square test. Comorbidities and demographic data that were significantly different between groups by chi-square analysis (p<0.05) were included in step-forward multivariable logistic regression. Variables included in multivariable analysis were anemia, atrial fibrillation, chronic kidney disease, hypertension, hyperlipidemia, heart failure, chronic obstructive pulmonary disease (COPD), coronary artery disease, diabetes mellitus, tobacco use, age group, race, payer, and year of admission. Multivariable odds ratios (OR) and 95% confidence intervals (CI) for marijuana use and other significant predictors were determined. Additionally, we conducted subgroup analysis of patients ≥ 70 years of age, those with reported tobacco use, and those with reported polysubstance use, accounting for the same covariates as in the primary analysis (S1 Fig). A p-value < 0.05 was considered significant throughout. All statistical analyses were performed with IBM SPSS Statistics (Version 22.0, IBM, Armonk, NY) and the R Statistical Package (Version 3.3.0, R Foundation for Statistical Computing, Vienna, Austria).

## Results

**Fig 2:**
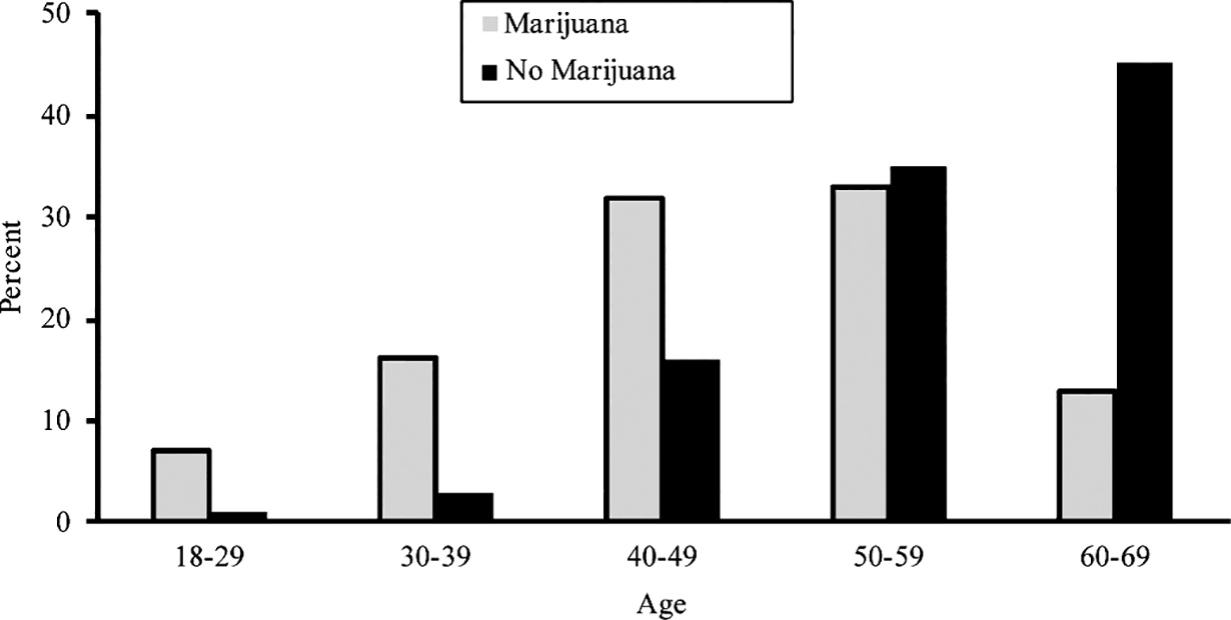
Incidence of AMI stratified by age and marijuana use.

The characteristics of the final study population stratified by marijuana use are shown in Table 1. A total of 3,854 out of 1,273,897 patients in the final data set reported marijuana use (approximately 1 in 331). Marijuana-using patients were more likely to be younger and male when compared to the control group (p<0.001 for both) with a mean age of 57.2 in the non-marijuana group and 47.2 years in the marijuana-using cohort. Incidence of AMI stratified by age and marijuana use is depicted in Fig 2. The incidence of AMI was higher in the cannabis-use group less than 50 years old when compared with non-users (54.0% vs 20.2%, p< 0.001). Both cohorts had a Caucasian majority, however the marijuana cohort included a larger percentage of African Americans (p*<*0.001). Patients in the marijuana group were more likely to be covered by Medicaid, county/indigent payers, or self-pay (p<0.001). They were also more likely to smoke tobacco, have COPD, hyperlipidemia, and chronic kidney disease *(*p<0.001 for all). However, they had a lower incidence of hypertension, heart failure, coronary artery disease, diabetes, and atrial fibrillation. Average length of stay for marijuana users was shorter than non-marijuana users (4.51 days vs. 6.25 days, respectively).

**Table 1:** Baseline characteristics, marijuana use vs. no marijuana use.

	Marijuana Users	Controls	P-value
	N = 3854	N = 1270043	
**Age, years‡**			**<0.001**
18–29	254 (7)*	6553 (1)	
30–39	609 (16)	43347 (3)	
40–49	1218 (32)	207062 (16)	
50–59	1267 (33)	438713 (35)	
60–69	506 (13)	574368 (45)	
**Race‡**			**<0.001**
White	1949 (51)	766554 (60)	
AA	804 (21)	118214 (9)	
Hispanic	367 (10)	146644 (12)	
Asian	14 (0)	28428 (2)	
Other	126 (3)	60038 (5)	
NA	594 (15)	150165 (12)	
**Gender‡**			**<0.001**
Male	2916 (76)	838727 (66)	
Female	747 (19)	401356 (32)	
NA	191 (5)	22960 (2)	
**Payer‡**			**<0.001**
Medicare	583 (15)	363007 (29)	
Medicaid	804 (21)	128636 (10)	
Private	1478 (38)	604604 (48)	
Workers comp	6 (0)	3760 (0)	
County/indigent	214 (6)	16875 (1)	
Other Government	65(2)	13493(1)	
Self-pay	661 (17)	91417 (7)	
Other	39 (1)	33670 (3)	
NA	4 (0)	14581 (1)	
**Comorbidities**			
Anemia†	445 (12)	170101 (13)	0.001
Hypertension‡	2046 (53)	717510 (57)	<0.001
Hyperlipidemia†	1676 (43)	519186 (41)	0.001
Heart failure‡	588 (15)	292133 (23)	<0.001
COPD‡	621 (16)	173697 (14)	<0.001
CKD‡	244 (6)	62671 (5)	<0.001
CAD‡	2384 (62)	819925 (65)	<0.001
Atrial fib‡	173 (5)	110153 (9)	<0.001
DM‡	746 (19)	411465 (32)	<0.001
Tobacco use‡	2256 (59)	337420 (27)	<0.001
**MI Type**			**0.001**
STEMI	1803 (47)	627095 (49)	
NSTEMI	2015 (53)	642948 (51)	

*Data are presented N (%) of patients unless otherwise indicated

Abbreviations: AA, African Ancestry; COPD, chronic obstructive pulmonary disease; CKD, chronic kidney disease; CAD, Coronary artery disease; A-fib, atrial fibrillation; DM, diabetes mellitus

*p< = 0.05

†p<0.01

‡p<0.001

**Fig 3:**
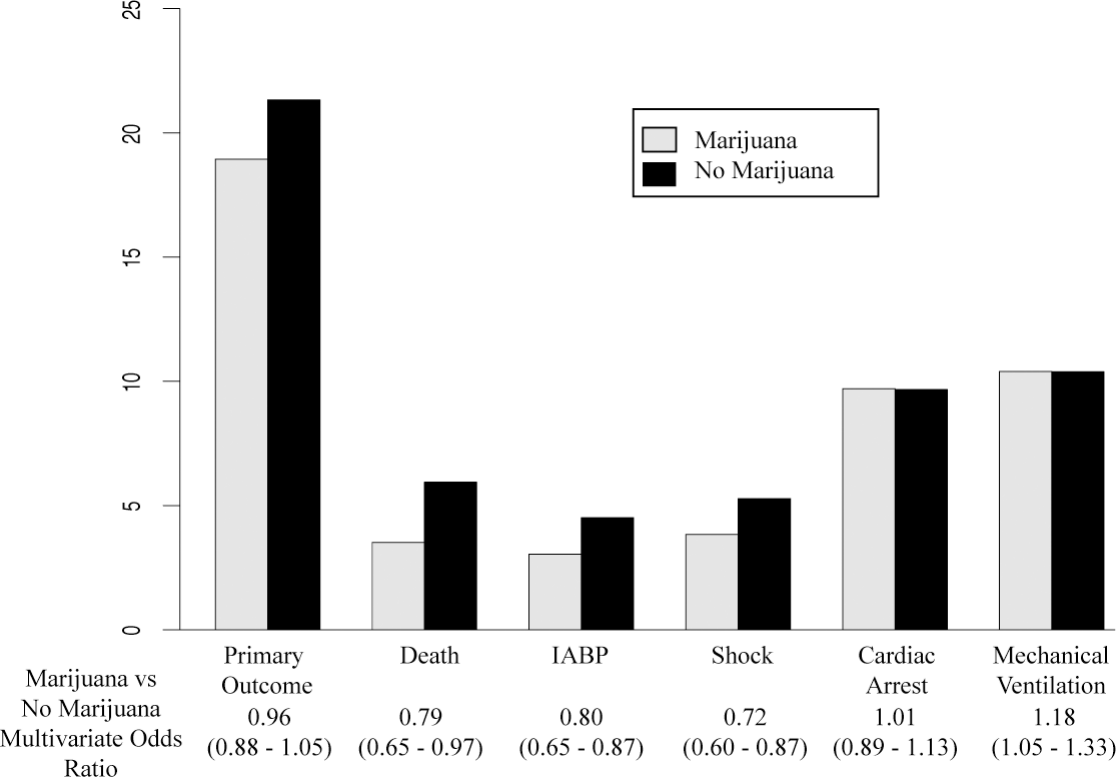
Rates of adverse outcomes in patients with acute myocardial infarction reported cannabis use vs. no cannabis use. Patients who reported marijuana use has decreased short-term risk of death, shock, and IABP placement in hospital while they had an increased risk of mechanical ventilation when compared to patients that reportedly did not use marijuana prior to admission for AMI.

**Fig 4:**
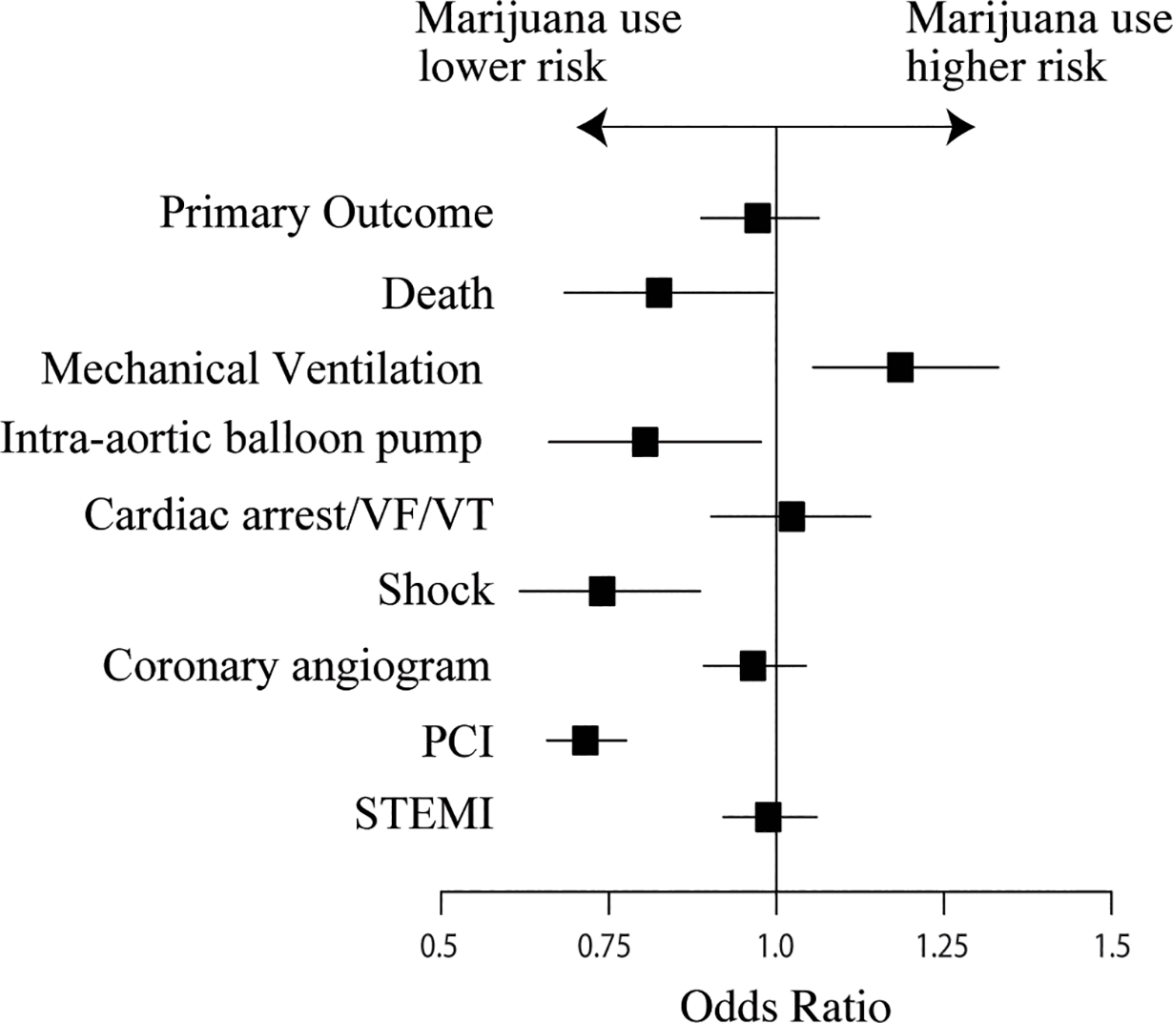
Multivariable odds ratios of each outcome associated with marijuana in final study population. Abbreviations: PCI, Percutaneous coronary intervention; VF, Ventricular fibrillation; VT, Ventricular tachycardia.

In total, 730 (18.9%) patients experienced the primary outcome in the marijuana cohort compared with 270,841 (21.3%) in the control cohort (p<0.001). By multivariable analysis, marijuana use was not significantly associated with the primary outcome (OR 0.97 [95% CI, 0.89 to 1.06; p = 0.53). In terms of secondary outcomes, marijuana use was associated with lower odds of mortality (OR, 0.79 [95% CI, 0.65 to 0.96]; p = 0.016), IABP placement (OR 0.82 [95% CI, 0.67 to 1.00]; p = 0.052), and shock (OR 0.72 [95% CI, 0.60 to 0.87] p<0.001). However, the marijuana group had an increased risk of requiring mechanical ventilation (OR 1.16 [95% CI, 1.02 to 1.30]; p = 0.015) post AMI. There was no increased risk of cardiac arrest in the marijuana cohort (OR 1.02 [95% CI 0.90–1.14]; p = 0.81). Although the odds of undergoing coronary angiography were similar between groups (OR 0.95 [95% CI 0.88 to 1.03]; p = 0.25), the marijuana-using group was significantly less likely to undergo percutaneous coronary intervention (PCI) (OR 0.73 [95% CI 0.67 to 0.80]; p<0.001). Rates of adverse outcomes are depicted in Table 2 and Figs 3 and 4.

**Table 2:** Outcomes in marijuana users vs. controls.

Outcome	Marijuana Users	Controls	Multivariable OR (95% CI)	p- value
**Composite Primary outcome**	730 (19)	270841 (21)	0.97 (0.89–1.06)	0.53
**Secondary outcomes**				
Death	135 (4)	75311 (6)	0.79 (0.65–0.96)	0.016
Shock	148 (4)	67048 (5)	0.72 (0.60–0.87)	<0.001
Mechanical ventilation	401 (10)	131835 (10)	1.16 (1.02–1.30	0.015
IABP	117 (3)	57292 (5)	0.82 (0.67–1.00)	0.052
Cardiac arrest	374 (10)	122797 (10)	1.015 (0.903–1.14)	0.81
Coronary angiogram	2204 (57)	663074 (52)	0.95 (0.88–1.03)	0.25
Coronary PCI	1355 (35)	401371 (32)	0.73 (0.67–0.80)	<0.001

IABP, intraaortic balloon pump; Coronary PCI, Coronary percutaneous intervention.

### Age specific analysis

To more specifically examine the effect of age on outcomes, the study population was organized into five age groups as shown in Table 1. We performed multivariable analysis within each age group separately in order to determine outcomes for each (S2 Fig). Similar to the whole cohort, there was no significant difference in the rate of primary outcome for marijuana users when compared to the control group in any single age group. However, patients who reported marijuana use in the age group 50–59 had a markedly decreased risk of mortality when compared to controls (OR 0.65 [95% CI 0.45 to 0.93]; p = 0.017). Marijuana users in the age groups 50–59 and 60–69 had a decreased risk of experiencing post-AMI cardiogenic shock. Patients in the age group 40–49 had a statistically significant increased risk of cardiac arrest (OR 1.27 [95% CI 0.71 to 1.33]; p = 0.02). Otherwise, there was no significant difference in risk of mortality, shock, or cardiac arrest between marijuana users and controls in the other age groups. Consistent with the primary analysis, each age group except for the youngest population (age 18–29) had a similar risk of cardiac angiogram but a decreased risk of PCI when compared to the control group.

### Impact of tobacco use

The marijuana cohort had a much higher incidence of concomitant tobacco use than the non-marijuana cohort (59% vs 27%, respectively). Therefore, in order to assess the potentially confounding effect of tobacco on outcomes found in this study, we separated tobacco and non-tobacco users, allowing us to analyze the impact of marijuana use in both cohorts. Regardless of tobacco use status, reported marijuana use prior to admission was associated with a higher risk of requiring mechanical ventilation. However, the risk of mechanical ventilation was significantly higher in patients who reported the use of both tobacco and marijuana (OR 1.71, [95% CI, 1.46 to 2.01]; p<0.001) compared to those who reported marijuana use alone (OR 1.23, [95% CI, 1.04 to 1.46]; p = 0.017).

## Discussion

We present the largest retrospective analysis of indexed admission AMI outcomes in marijuana users. The major findings of this study are: (1) mean age of admission for AMI was on average 10 years younger in marijuana users vs. non-users; (2) patients with reported marijuana use had a lower likelihood of in-hospital mortality compared with patients without reported marijuana use; and (3) patients who had used marijuana were more likely to be placed on mechanical ventilation during their hospital stay when compared to non-marijuana users.

Perhaps the most striking finding of our study is that marijuana use prior to AMI was associated with decreased in-hospital mortality post AMI. Interestingly, Desai et al [13], also found a significant *decrease* in mortality following AMI in marijuana users (OR 0.742, CI 0.693–0.795). However, the authors report simply that there was no increase in the odds of mortality in marijuana users following AMI. The similarities between the populations studied in this analysis and the study by Desai, et al [13] are striking: the marijuana users admitted with AMI were younger, male, African American with decreased incidence of hypertension, heart failure, diabetes and arrhythmias, with an increase in COPD and tobacco use. Given the similarities in population characteristics as well as in outcome measures, we would strongly suggest that marijuana use is associated with a significant decrease in in-hospital mortality rather than no increase in mortality.

There are a few possible explanations for this result. Importantly, it is critical to consider the younger age, decreased incidence of known cardiac risk factors, and increased tobacco use within the marijuana cohort, as these are potential confounding variables. In previous studies, Mittleman demonstrated the mean age of marijuana users that experienced a myocardial infarction was 48±8 years, compared with 62±13 years for non-users [15], and Desai found mean ages of AMI patients to be 49±11 and 58±9 in marijuana users versus non-users, consistent with what we found between users and non-users (47.2 versus 57.2 respectively) in our study. However, age-specific analysis and controlling for other potential confounders did not explain these findings in either our study or the previous study [13].

The protective effect we found on short-term outcomes post AMI could also be similar to the “smoker’s paradox,” referring to the observed short-term mortality benefit in tobacco users post AMI [16, 17]. Several studies over the past 20 years have described a mortality benefit conferred in tobacco users in the 30-day period post AMI, especially with use of thrombolytic therapy for treatment [18, 19]. Additionally, a “dose response” relationship has been described in the era of thrombolytic therapy for AMI, with the in-hospital mortality rate increasing from 2.3–4.7% in smokers, to 5.2–7.6% in former smokers, and to 7.0–13.8% in non-smokers [20]. In the era of PCI, the CADILLAC trial showed that the mortality benefit for tobacco users persisted for both 30-days and 1 year post AMI, and the dose dependent relationship between mortality benefit and current, former, and never smoking status was still present [17]. However, the mortality benefit at 1 year did not persist in multivariable analysis, when all of the population baseline characteristics were taken into consideration [17], as the population that used tobacco was 10 years younger on average and had significantly decreased rates of several comorbidities that have a negative effect on prognosis. This is similar to our population, such that the marijuana users were younger and had a significantly lower percentage of several important cardiac risk factors. Thus, it is entirely possible that we are observing an effect similar to the “smoker’s paradox” seen in tobacco users.

Another possible explanation for the mortality benefit seen in this study is that marijuana use may have provided a cardioprotective effect to users. While small clinical studies have previously suggested worse AMI outcomes associated with marijuana use [12], there is evidence that marijuana could be protective in the setting of myocardial ischemia [21–23]. In a study by Montecucco, a single dose of CB2 agonist administered during ischemia reduced the infarct size following reperfusion [22]. Decreased infiltration of inflammatory cells has been observed post-MI in mice that were pretreated with low dose THC [23], and other studies have the noted protective effects of CB2 receptor agonism against reperfusion injury and ischemia induced cardiac arrhythmias [21, 24, 25]. Furthermore, exposure to cannabinoids has been shown to decrease the area of necrosis during ischemia-reperfusion [24] and decrease the incidence of ischemia-reperfusion arrhythmias [24]. The observed outcomes of lower rates of mortality, shock, and IABP placement in this analysis could be the result of cardioprotective effects of marijuana during AMI conferred by activation of CB2 receptors [21–23].

Lastly, marijuana use may be associated with an increased incidence of smaller, non-fatal AMIs (in a younger population that may not have otherwise experienced a cardiac event). In univariate analysis, marijuana use was associated with an increase incidence of NSTEMI (53%) compared to no marijuana use (51%). However, marijuana use was not significantly associated with STEMI vs. NSTEMI in multivariable analysis (OR 1.00 [0.93–1.08], p = 0.96, Fig 4). Nonetheless, we compared STEMI and NSTEMI outcomes separately (S3 Fig), and found that marijuana use was associated with significantly lower odds of death in patients admitted with STEMI (OR 0.76 [0.58–0.98]) whereas there was no association between marijuana use and mortality in the NSTEMI group. In both STEMI and NSTEMI groups, patients with marijuana use were less likely to receive PCI than patients who did not use marijuana.

Several studies have indicated that smoking marijuana is associated with a dose dependent increase in heart rate [26–28]. Additionally, smoking marijuana decreases oxygen carrying capacity in the face of an increased oxygen demand. Aronow and Cassidy [29] demonstrated that among people with stable angina, smoking one marijuana cigarette decreases the time to angina with exercise by 48% compared with a 9% decrease in subjects smoking a marijuana placebo that did not contain delta-9-tetrahydrocannabinol. In this analysis, we found that marijuana use was associated with similar risk of receiving a coronary angiogram when compared to the control group, but marijuana-use patients were significantly less likely to undergo PCI. This suggests that these marijuana-use patients did not have significant coronary artery disease or acute plaque rupture as the inciting event for their AMI. Several case reports have suggested that vasospasm or acute thrombosis may represent a primary mechanism of AMI in marijuana users [30]. Each of these details must be taken into account when considering the results of this study.

Mechanical ventilation was the only outcome that demonstrated increased risk in patients that had used marijuana prior to admission for AMI. Subgroup analysis revealed that while marijuana use alone increased risk of mechanical ventilation, concomitant use of tobacco and marijuana were correlated with a substantially increased risk in comparison. There could be a number of explanations for this association. Although there is no data available on the method by which patients used marijuana, smoking remains the most common method of use. Smoking cannabis has been shown to have similar respiratory effects as smoking crack cocaine, such as severe bullous emphysema [27]. In addition to acute respiratory insults, smoking marijuana early in life may be an important risk factor for the development of obstructive airway disease later in life [26], and a follow-up study suggested a progressive decline in FEV1/FVC ratio with continuous marijuana smoking [28]. In the present analysis, a large percentage of the marijuana cohort also reported tobacco use, a known risk factor emphysema and chronic bronchitis. Considering the increased risk of mechanical ventilation post AMI, tobacco use and marijuana use may have an additive effect in our study.

### Study limitations

The present study has several potential limitations characteristic to retrospective studies using administrative data. Because all data were de-identified, patient level data were not available to verify coding accuracy. Angiograms, laboratory tests, medications taken pre- or post-AMI, and vital signs on admission were not available. Furthermore, there were no post-discharge data including long-term mortality and readmissions. The basis for a diagnosis of marijuana use was not known, and it is highly likely that marijuana use was significantly underreported. Therefore, the control group may have had a substantial number of marijuana users in it, which could have unpredictable effects on statistical results, although the large number of controls likely mitigated these effects. The route, amount and frequency of marijuana use in each patient could not be determined, so a dose-response effect could not be established. Additionally, the time since last use could not be determined so it is possible that self-reported use was quite distant to the cardiac event and had little or no impact on the initiation of AMI or on the short-term outcomes.

## Conclusion

In this large, multiregional analysis, marijuana use reported during hospitalization for AMI was associated with a significantly decreased risk of in-hospital mortality, IABP placement, and shock, and a significantly increased risk of mechanical ventilation post AMI. Given the increasing prevalence and acceptance of marijuana use, these findings suggest that additional study is warranted to further investigate these discoveries and to identify potential mechanisms by which marijuana is associated with improved short-term outcomes following AMI and for mitigating the possible negative effects of concomitant substance use.

## Supporting information

S1 Table Sources of de-identified data. (DOCX)

S2 Table ICD-9 CM codes for comorbid conditions. (DOCX)

S3 Table Patient characteristics of the original study population. (DOCX)

S1 Fig Outcomes in patients with concurrent cardiotoxic substance use and in patients > 70 years of age. (DOCX)

S2 Fig Age-specific analysis of outcomes. Primary outcome includes: in-hospital death, intraaortic balloon pump placement, (IABP), mechanical ventilation, cardiac arrest, and shock.
(DOCX)

S3 Fig Analysis of outcomes in STEMI and NSTEMI patients. Primary outcome includes: in-hospital death, intraaortic balloon pump placement, (IABP), mechanical ventilation, cardiac arrest, and shock.
(DOCX)

## Abbreviations

AMI: acute myocardial infarction
IABP: Intraaortic balloon pump
OR: odds ratio
CI: confidence interval
NSTEMI: non-ST-segment elevation myocardial infarction
PCI: percutaneous coronary intervention
STEMI: ST-segment elevation myocardial infarction

## References

[1] World Health Organization 2015 [Available from: http://www.who.int/substance_abuse/facts/cannabis/en/.

[2] Ingraham C. How many Americans regularly use pot? The number is, errr, higher than you think. Sacramento Bee [Internet]. 2017 Available from: http://www.sacbee.com/news/nation-world/national/article145681414.html—storylink=cpy.

[3] National Institute on Drug Abuse National Institute on Drug Abuse2015 [Available from: http://www.drugabuse.gov/publications/drugfacts/nationwide-trends.

[4] Iversen LL. Pharmacology. Medical uses of marijuana? Nature. 1993;365(6441):12–3. doi: 10.1038/365012a0 8395659 10.1038/365012a0

[5] Arora S, Goyal H, Aggarwal P, Kukar A. ST-segment elevation myocardial infarction in a 37-year-old man with normal coronaries—it is not always cocaine! The American journal of emergency medicine. 2012;30(9):2091 e3-5. 10.1016/j.ajem.2011.12.033 22306387

[6] Basnet S, Mander G, Nicolas R. Coronary vasospasm in an adolescent resulting from marijuana use. Pediatric cardiology. 2009;30(4):543–5. 10.1007/s00246-009-9384-7 19212697

[7] Rezkalla SH, Sharma P, Kloner RA. Coronary no-flow and ventricular tachycardia associated with habitual marijuana use. Ann Emerg Med. 2003;42(3):365–9. doi: 10.1067/mem.2003.297 12944889 10.1016/s0196-0644(03)00426-8

[8] Rodondi N, Pletcher MJ, Liu K, Hulley SB, Sidney S, Coronary Artery Risk Development in Young Adults S. Marijuana use, diet, body mass index, and cardiovascular risk factors (from the CARDIA study). Am J Cardiol. 2006;98(4):478–84. doi: 10.1016/j.amjcard.2006.03.024 16893701 10.1016/j.amjcard.2006.03.024

[9] Jouanjus E, Lapeyre-Mestre M, Micallef J, French Association of the Regional A, Dependence Monitoring Centres Working Group on Cannabis C. Cannabis use: signal of increasing risk of serious cardiovascular disorders. J Am Heart Assoc. 2014;3(2):e000638 doi: 10.1161/JAHA.113.000638 24760961 10.1161/JAHA.113.000638 PMC4187498

[10] Dhingra R, Vasan RS. Age as a risk factor. Med Clin North Am. 2012;96(1):87–91. doi: 10.1016/j.mcna.2011.11.003 22391253 10.1016/j.mcna.2011.11.003 PMC3297980

[11] Frost L, Mostofsky E, Rosenbloom JI, Mukamal KJ, Mittleman MA. Marijuana use and long-term mortality among survivors of acute myocardial infarction. Am Heart J. 2013;165(2):170–5. doi: 10.1016/j.ahj.2012.11.007 23351819 10.1016/j.ahj.2012.11.007 PMC3558923

[12] Mukamal KJ, Maclure M, Muller JE, Mittleman MA. An exploratory prospective study of marijuana use and mortality following acute myocardial infarction. Am Heart J. 2008;155(3):465–70. doi: 10.1016/j.ahj.2007.10.049 18294478 10.1016/j.ahj.2007.10.049 PMC2276621

[13] Desai R, Patel U, Sharma S, Amin P, Bhuva R, Patel MS, et al Recreational Marijuana Use and Acute Myocardial Infarction: Insights from Nationwide Inpatient Sample in the United States. Cureus. 2017;9(11):e1816 doi: 10.7759/cureus.1816 29312837 10.7759/cureus.1816 PMC5752226

[14] Celermajer DS, Sholler GF, Howman-Giles R, Celermajer JM. Myocardial infarction in childhood: clinical analysis of 17 cases and medium term follow up of survivors. British heart journal. 1991;65(6):332–6. 2054243 10.1136/hrt.65.6.332 PMC1024677

[15] Mittleman MA, Lewis RA, Maclure M, Sherwood JB, Muller JE. Triggering myocardial infarction by marijuana. Circulation. 2001;103(23):2805–9. 11401936 10.1161/01.cir.103.23.2805

[16] Bell TM, Bayt DR, Zarzaur BL. "Smoker's Paradox" in Patients Treated for Severe Injuries: Lower Risk of Mortality After Trauma Observed in Current Smokers. Nicotine Tob Res. 2015;17(12):1499–504. doi: 10.1093/ntr/ntv027 25646350 10.1093/ntr/ntv027 PMC4757929

[17] Gupta T, Kolte D, Khera S, Harikrishnan P, Mujib M, Aronow WS, et al Smoker's Paradox in Patients With ST-Segment Elevation Myocardial Infarction Undergoing Primary Percutaneous Coronary Intervention. J Am Heart Assoc. 2016;5(4). 10.1161/JAHA.116.003370 PMC4843594 27107131

[18] Aune E, Roislien J, Mathisen M, Thelle DS, Otterstad JE. The "smoker's paradox" in patients with acute coronary syndrome: a systematic review. BMC Med. 2011;9:97 doi: 10.1186/1741-7015-9-97 21861870 10.1186/1741-7015-9-97 PMC3179733

[19] Grines CL, Topol EJ, O'Neill WW, George BS, Kereiakes D, Phillips HR, et al Effect of cigarette smoking on outcome after thrombolytic therapy for myocardial infarction. Circulation. 1995;91(2):298–303. 7805231 10.1161/01.cir.91.2.298

[20] Bastos-Amador P, Almendro-Delia M, Munoz-Calero B, Blanco-Ponce E, Recio-Mayoral A, Reina-Toral A, et al The tobacco paradox in acute coronary syndrome. The prior cessation of smoking as a marker of a better short-term prognosis. Rev Clin Esp. 2016;216(6):301–7. doi: 10.1016/j.rce.2016.03.006 27118137 10.1016/j.rce.2016.03.006

[21] Defer N, Wan J, Souktani R, Escoubet B, Perier M, Caramelle P, et al The cannabinoid receptor type 2 promotes cardiac myocyte and fibroblast survival and protects against ischemia/reperfusion-induced cardiomyopathy. FASEB J. 2009;23(7):2120–30. doi: 10.1096/fj.09-129478 19246487 10.1096/fj.09-129478

[22] Montecucco F, Lenglet S, Braunersreuther V, Burger F, Pelli G, Bertolotto M, et al CB(2) cannabinoid receptor activation is cardioprotective in a mouse model of ischemia/reperfusion. J Mol Cell Cardiol. 2009;46(5):612–20. doi: 10.1016/j.yjmcc.2008.12.014 19162037 10.1016/j.yjmcc.2008.12.014

[23] Waldman M, Hochhauser E, Fishbein M, Aravot D, Shainberg A, Sarne Y. An ultra-low dose of tetrahydrocannabinol provides cardioprotection. Biochem Pharmacol. 2013;85(11):1626–33. doi: 10.1016/j.bcp.2013.03.014 23537701 10.1016/j.bcp.2013.03.014

[24] Krylatov AV, Ugdyzhekova DS, Bernatskaya NA, Maslov LN, Mekhoulam R, Pertwee RG, et al Activation of type II cannabinoid receptors improves myocardial tolerance to arrhythmogenic effects of coronary occlusion and reperfusion. Bull Exp Biol Med. 2001;131(6):523–5. 11586395 10.1023/a:1012381914518

[25] Shmist YA, Goncharov I, Eichler M, Shneyvays V, Isaac A, Vogel Z, et al Delta-9-tetrahydrocannabinol protects cardiac cells from hypoxia via CB2 receptor activation and nitric oxide production. Mol Cell Biochem. 2006;283(1–2):75–83. doi: 10.1007/s11010-006-2346-y 16444588 10.1007/s11010-006-2346-y

[26] Bloom JW, Kaltenborn WT, Paoletti P, Camilli A, Lebowitz MD. Respiratory effects of non-tobacco cigarettes. Br Med J (Clin Res Ed). 1987;295(6612):1516–8. 10.1136/bmj.295.6612.1516 PMC1248665 3122882

[27] Devlin RJ, Henry JA. Clinical review: Major consequences of illicit drug consumption. Crit Care. 2008;12(1):202 doi: 10.1186/cc6166 18279535 10.1186/cc6166 PMC2374627

[28] Sherrill DL, Krzyzanowski M, Bloom JW, Lebowitz MD. Respiratory effects of non-tobacco cigarettes: a longitudinal study in general population. Int J Epidemiol. 1991;20(1):132–7. 2066211 10.1093/ije/20.1.132

[29] Aronow WS, Cassidy J. Effect of marihuana and placebo-marihuana smoking on angina pectoris. N Engl J Med. 1974;291(2):65–7. doi: 10.1056/NEJM197407112910203 4599385 10.1056/NEJM197407112910203

[30] Casier I, Vanduynhoven P, Haine S, Vrints C, Jorens PG. Is recent cannabis use associated with acute coronary syndromes? An illustrative case series. Acta Cardiol. 2014;69(2):131–6. doi: 10.2143/AC.69.2.3017293 24783463 10.1080/ac.69.2.3017293

