# Supplementary material for: Marijuana use and short-term outcomes in patients hospitalized for acute myocardial infarction
Source: PLoS One. 2018 Jul 11;13(7):e0199705. doi: 10.1371/journal.pone.0199705 (PMC6040751; doi:10.1371/journal.pone.0199705)
Supplement: S1 Table — (DOCX) [file pone.0199705.s001.docx]

**S1 Table. Sources of de-identified data.**

| State | Agency | URL | Access Date |
| --- | --- | --- | --- |
| CA | California Office of Statewide Health Plannning and Development | [http://www.oshpd.ca.gov/HID](http://www.oshpd.ca.gov/HID/Products/PatDischargeData/PublicDataSet/index.html)/ | March 20, 2013 |
| NY | New York Department of Health, Statewide Planning and Research Cooerative System (SPARCS) | <http://www.health.ny.gov/statistics/sparcs/> | August 16, 2013 |
| NJ | New Jersey Department of Health, Office of Health Care Quality Assessment | <http://www.state.nj.us/health/healthcarequality> | August 16, 2013 |
| VT | Vermont Department of Health | http://www.healthvermont.gov/health-statistics-vital-records/health-care-systems-reporting/hospital-discharge-data | August 16, 2013 |
| NH | New Hampshire Department of Health and Human Services | <http://www.dhhs.nh.gov/dphs/hsdm/requests.htm> | March 20, 2013 |
| TX | Texas Department of State Health Services. | http://healthdata.dshs.texas.gov/Home | June 28, 2015 |
| WV | West Virginia Health Care Authority | <http://www.hca.wv.gov/data/requestdata/pages/default.aspx> | May 21, 2013 |
| CO | Colorado Hospital Association | <http://www.cha.com/> | March 20, 2015 |
