# Supplementary material for: Marijuana use and short-term outcomes in patients hospitalized for acute myocardial infarction
Source: PLoS One. 2018 Jul 11;13(7):e0199705. doi: 10.1371/journal.pone.0199705 (PMC6040751; doi:10.1371/journal.pone.0199705)
Supplement: S2 Table — (DOCX) [file pone.0199705.s002.docx]

**S2 Table. ICD-9 CM Codes for Comorbid Conditions**

| dxmapid | dxcode | dxtext | dxcui | dxpoa |
| --- | --- | --- | --- | --- |
| 1 | 3052 | Cannabis | NULL | NULL |
| 2 | 30520 | Cannabis | NULL | NULL |
| 3 | 30521 | Cannabis | NULL | NULL |
| 4 | 30522 | Cannabis | NULL | NULL |
| 5 | 30523 | Cannabis | NULL | NULL |
| 6 | 3043 | Cannabis | NULL | NULL |
| 7 | 30430 | Cannabis | NULL | NULL |
| 8 | 30431 | Cannabis | NULL | NULL |
| 9 | 30432 | Cannabis | NULL | NULL |
| 10 | 30433 | Cannabis | NULL | NULL |
| 11 | 2720 | Hyperlipidemia | NULL | NULL |
| 12 | 2721 | Hyperlipidemia | NULL | NULL |
| 13 | 2722 | Hyperlipidemia | NULL | NULL |
| 14 | 2724 | Hyperlipidemia | NULL | NULL |
| 15 | 3051 | Tobacco Use | NULL | NULL |
| 16 | 98984 | Tobacco Use | NULL | NULL |
| 17 | V1582 | Tobacco Use | NULL | NULL |
| 18 | 250 | Diabetes mellitus | NULL | NULL |
| 19 | 2500 | Diabetes mellitus | NULL | NULL |
| 20 | 25000 | Diabetes mellitus | NULL | NULL |
| 21 | 25001 | Diabetes mellitus | NULL | NULL |
| 22 | 25002 | Diabetes mellitus | NULL | NULL |
| 23 | 25003 | Diabetes mellitus | NULL | NULL |
| 24 | 2501 | Diabetes mellitus | NULL | NULL |
| 25 | 25010 | Diabetes mellitus | NULL | NULL |
| 26 | 25011 | Diabetes mellitus | NULL | NULL |
| 27 | 25012 | Diabetes mellitus | NULL | NULL |
| 28 | 25013 | Diabetes mellitus | NULL | NULL |
| 29 | 2502 | Diabetes mellitus | NULL | NULL |
| 30 | 25020 | Diabetes mellitus | NULL | NULL |
| 31 | 25021 | Diabetes mellitus | NULL | NULL |
| 32 | 25022 | Diabetes mellitus | NULL | NULL |
| 33 | 25023 | Diabetes mellitus | NULL | NULL |
| 34 | 2503 | Diabetes mellitus | NULL | NULL |
| 35 | 25030 | Diabetes mellitus | NULL | NULL |
| 36 | 25031 | Diabetes mellitus | NULL | NULL |
| 37 | 25032 | Diabetes mellitus | NULL | NULL |
| 38 | 25033 | Diabetes mellitus | NULL | NULL |
| 39 | 2504 | Diabetes mellitus | NULL | NULL |
| 40 | 25040 | Diabetes mellitus | NULL | NULL |
| 41 | 25041 | Diabetes mellitus | NULL | NULL |
| 42 | 25042 | Diabetes mellitus | NULL | NULL |
| 43 | 25043 | Diabetes mellitus | NULL | NULL |
| 44 | 2505 | Diabetes mellitus | NULL | NULL |
| 45 | 25050 | Diabetes mellitus | NULL | NULL |
| 46 | 25051 | Diabetes mellitus | NULL | NULL |
| 47 | 25052 | Diabetes mellitus | NULL | NULL |
| 48 | 25053 | Diabetes mellitus | NULL | NULL |
| 49 | 2506 | Diabetes mellitus | NULL | NULL |
| 50 | 25061 | Diabetes mellitus | NULL | NULL |
| 51 | 25062 | Diabetes mellitus | NULL | NULL |
| 52 | 25063 | Diabetes mellitus | NULL | NULL |
| 53 | 2507 | Diabetes mellitus | NULL | NULL |
| 54 | 25071 | Diabetes mellitus | NULL | NULL |
| 55 | 25072 | Diabetes mellitus | NULL | NULL |
| 56 | 25073 | Diabetes mellitus | NULL | NULL |
| 57 | 25081 | Diabetes mellitus | NULL | NULL |
| 58 | 25082 | Diabetes mellitus | NULL | NULL |
| 59 | 2508 | Diabetes mellitus | NULL | NULL |
| 60 | 25083 | Diabetes mellitus | NULL | NULL |
| 61 | 2509 | Diabetes mellitus | NULL | NULL |
| 62 | 25090 | Diabetes mellitus | NULL | NULL |
| 63 | 25091 | Diabetes mellitus | NULL | NULL |
| 64 | 25092 | Diabetes mellitus | NULL | NULL |
| 65 | 25093 | Diabetes mellitus | NULL | NULL |
| 66 | 249 | Diabetes mellitus | NULL | NULL |
| 67 | 2490 | Diabetes mellitus | NULL | NULL |
| 68 | 24900 | Diabetes mellitus | NULL | NULL |
| 69 | 24901 | Diabetes mellitus | NULL | NULL |
| 70 | 2491 | Diabetes mellitus | NULL | NULL |
| 71 | 24910 | Diabetes mellitus | NULL | NULL |
| 72 | 24911 | Diabetes mellitus | NULL | NULL |
| 73 | 2492 | Diabetes mellitus | NULL | NULL |
| 74 | 24920 | Diabetes mellitus | NULL | NULL |
| 75 | 24921 | Diabetes mellitus | NULL | NULL |
| 76 | 2493 | Diabetes mellitus | NULL | NULL |
| 77 | 24930 | Diabetes mellitus | NULL | NULL |
| 78 | 24931 | Diabetes mellitus | NULL | NULL |
| 79 | 2494 | Diabetes mellitus | NULL | NULL |
| 80 | 24940 | Diabetes mellitus | NULL | NULL |
| 81 | 24941 | Diabetes mellitus | NULL | NULL |
| 82 | 2495 | Diabetes mellitus | NULL | NULL |
| 83 | 24950 | Diabetes mellitus | NULL | NULL |
| 84 | 24951 | Diabetes mellitus | NULL | NULL |
| 85 | 2496 | Diabetes mellitus | NULL | NULL |
| 86 | 24960 | Diabetes mellitus | NULL | NULL |
| 87 | 24961 | Diabetes mellitus | NULL | NULL |
| 88 | 2497 | Diabetes mellitus | NULL | NULL |
| 89 | 24970 | Diabetes mellitus | NULL | NULL |
| 90 | 24971 | Diabetes mellitus | NULL | NULL |
| 91 | 2498 | Diabetes mellitus | NULL | NULL |
| 92 | 24980 | Diabetes mellitus | NULL | NULL |
| 93 | 24981 | Diabetes mellitus | NULL | NULL |
| 94 | 2499 | Diabetes mellitus | NULL | NULL |
| 95 | 24990 | Diabetes mellitus | NULL | NULL |
| 96 | 24991 | Diabetes mellitus | NULL | NULL |
| 97 | 401 | Hypertension | NULL | NULL |
| 98 | 402 | Hypertension | NULL | NULL |
| 99 | 403 | Hypertension | NULL | NULL |
| 100 | 404 | Hypertension | NULL | NULL |
| 101 | 405 | Hypertension | NULL | NULL |
| 102 | 4010 | Hypertension | NULL | NULL |
| 103 | 4011 | Hypertension | NULL | NULL |
| 104 | 4019 | Hypertension | NULL | NULL |
| 105 | 4020 | Hypertension | NULL | NULL |
| 106 | 40200 | Hypertension | NULL | NULL |
| 107 | 40201 | Hypertension | NULL | NULL |
| 108 | 4021 | Hypertension | NULL | NULL |
| 109 | 40210 | Hypertension | NULL | NULL |
| 110 | 40211 | Hypertension | NULL | NULL |
| 111 | 4029 | Hypertension | NULL | NULL |
| 112 | 40290 | Hypertension | NULL | NULL |
| 113 | 40291 | Hypertension | NULL | NULL |
| 114 | 4030 | Hypertension | NULL | NULL |
| 115 | 40300 | Hypertension | NULL | NULL |
| 116 | 40301 | Hypertension | NULL | NULL |
| 117 | 4031 | Hypertension | NULL | NULL |
| 118 | 40310 | Hypertension | NULL | NULL |
| 119 | 40311 | Hypertension | NULL | NULL |
| 120 | 4039 | Hypertension | NULL | NULL |
| 121 | 40390 | Hypertension | NULL | NULL |
| 122 | 40391 | Hypertension | NULL | NULL |
| 123 | 4040 | Hypertension | NULL | NULL |
| 124 | 40400 | Hypertension | NULL | NULL |
| 125 | 40401 | Hypertension | NULL | NULL |
| 126 | 40402 | Hypertension | NULL | NULL |
| 127 | 40403 | Hypertension | NULL | NULL |
| 128 | 4041 | Hypertension | NULL | NULL |
| 129 | 40410 | Hypertension | NULL | NULL |
| 130 | 40411 | Hypertension | NULL | NULL |
| 131 | 40412 | Hypertension | NULL | NULL |
| 132 | 40413 | Hypertension | NULL | NULL |
| 133 | 4049 | Hypertension | NULL | NULL |
| 134 | 40490 | Hypertension | NULL | NULL |
| 135 | 40491 | Hypertension | NULL | NULL |
| 136 | 40492 | Hypertension | NULL | NULL |
| 137 | 40493 | Hypertension | NULL | NULL |
| 138 | 4050 | Hypertension | NULL | NULL |
| 139 | 40501 | Hypertension | NULL | NULL |
| 140 | 40509 | Hypertension | NULL | NULL |
| 141 | 4051 | Hypertension | NULL | NULL |
| 142 | 40511 | Hypertension | NULL | NULL |
| 143 | 40519 | Hypertension | NULL | NULL |
| 144 | 4059 | Hypertension | NULL | NULL |
| 145 | 40591 | Hypertension | NULL | NULL |
| 146 | 40599 | Hypertension | NULL | NULL |
| 147 | 428 | Heart failure | NULL | NULL |
| 148 | 4280 | Heart failure | NULL | NULL |
| 149 | 4281 | Heart failure | NULL | NULL |
| 150 | 4282 | Heart failure | NULL | NULL |
| 151 | 42820 | Heart failure | NULL | NULL |
| 152 | 42821 | Heart failure | NULL | NULL |
| 153 | 42822 | Heart failure | NULL | NULL |
| 154 | 42823 | Heart failure | NULL | NULL |
| 155 | 4283 | Heart failure | NULL | NULL |
| 156 | 42830 | Heart failure | NULL | NULL |
| 157 | 42831 | Heart failure | NULL | NULL |
| 158 | 42832 | Heart failure | NULL | NULL |
| 159 | 42833 | Heart failure | NULL | NULL |
| 160 | 4284 | Heart failure | NULL | NULL |
| 161 | 42840 | Heart failure | NULL | NULL |
| 162 | 42841 | Heart failure | NULL | NULL |
| 163 | 42842 | Heart failure | NULL | NULL |
| 164 | 42843 | Heart failure | NULL | NULL |
| 165 | 4289 | Heart failure | NULL | NULL |
| 166 | 3000 | Anxiety | NULL | NULL |
| 167 | 30000 | Anxiety | NULL | NULL |
| 168 | 30001 | Anxiety | NULL | NULL |
| 169 | 30002 | Anxiety | NULL | NULL |
| 170 | 30009 | Anxiety | NULL | NULL |
| 171 | 29384 | Anxiety | NULL | NULL |
| 172 | 30924 | Anxiety | NULL | NULL |
| 173 | 30928 | Anxiety | NULL | NULL |
| 174 | 3002 | Anxiety | NULL | NULL |
| 175 | 30020 | Anxiety | NULL | NULL |
| 176 | 30021 | Anxiety | NULL | NULL |
| 177 | 30022 | Anxiety | NULL | NULL |
| 178 | 30023 | Anxiety | NULL | NULL |
| 179 | 30029 | Anxiety | NULL | NULL |
| 180 | 3004 | Depression | NULL | NULL |
| 181 | 2962 | Depression | NULL | NULL |
| 182 | 29620 | Depression | NULL | NULL |
| 183 | 29621 | Depression | NULL | NULL |
| 184 | 29622 | Depression | NULL | NULL |
| 185 | 29623 | Depression | NULL | NULL |
| 186 | 29624 | Depression | NULL | NULL |
| 187 | 29625 | Depression | NULL | NULL |
| 188 | 29626 | Depression | NULL | NULL |
| 189 | 2963 | Depression | NULL | NULL |
| 190 | 29631 | Depression | NULL | NULL |
| 191 | 29632 | Depression | NULL | NULL |
| 192 | 29633 | Depression | NULL | NULL |
| 193 | 29634 | Depression | NULL | NULL |
| 194 | 29635 | Depression | NULL | NULL |
| 195 | 29636 | Depression | NULL | NULL |
| 196 | 29690 | Depression | NULL | NULL |
| 197 | 2965 | Depression | NULL | NULL |
| 198 | 29650 | Depression | NULL | NULL |
| 199 | 29651 | Depression | NULL | NULL |
| 200 | 29652 | Depression | NULL | NULL |
| 201 | 29653 | Depression | NULL | NULL |
| 202 | 29654 | Depression | NULL | NULL |
| 203 | 29655 | Depression | NULL | NULL |
| 204 | 29656 | Depression | NULL | NULL |
| 205 | 29383 | Depression | NULL | NULL |
| 206 | 4140 | CAD | NULL | NULL |
| 207 | 41401 | CAD | NULL | NULL |
| 208 | 41402 | CAD | NULL | NULL |
| 209 | 41403 | CAD | NULL | NULL |
| 210 | 41404 | CAD | NULL | NULL |
| 211 | 41405 | CAD | NULL | NULL |
| 212 | 41406 | CAD | NULL | NULL |
| 213 | 41407 | CAD | NULL | NULL |
| 214 | 4142 | CAD | NULL | NULL |
| 215 | 4143 | CAD | NULL | NULL |
| 216 | 4148 | CAD | NULL | NULL |
| 217 | 4149 | CAD | NULL | NULL |
| 218 | 280 | Anemia | NULL | NULL |
| 219 | 2800 | Anemia | NULL | NULL |
| 220 | 2801 | Anemia | NULL | NULL |
| 221 | 2808 | Anemia | NULL | NULL |
| 222 | 2809 | Anemia | NULL | NULL |
| 223 | 281 | Anemia | NULL | NULL |
| 224 | 2810 | Anemia | NULL | NULL |
| 225 | 2811 | Anemia | NULL | NULL |
| 226 | 2812 | Anemia | NULL | NULL |
| 227 | 2813 | Anemia | NULL | NULL |
| 228 | 2814 | Anemia | NULL | NULL |
| 229 | 2818 | Anemia | NULL | NULL |
| 230 | 2819 | Anemia | NULL | NULL |
| 231 | 282 | Anemia | NULL | NULL |
| 232 | 2821 | Anemia | NULL | NULL |
| 233 | 2822 | Anemia | NULL | NULL |
| 234 | 2823 | Anemia | NULL | NULL |
| 235 | 2824 | Anemia | NULL | NULL |
| 236 | 28241 | Anemia | NULL | NULL |
| 237 | 28242 | Anemia | NULL | NULL |
| 238 | 28249 | Anemia | NULL | NULL |
| 239 | 2825 | Anemia | NULL | NULL |
| 240 | 2826 | Anemia | NULL | NULL |
| 241 | 28260 | Anemia | NULL | NULL |
| 242 | 28261 | Anemia | NULL | NULL |
| 243 | 28262 | Anemia | NULL | NULL |
| 244 | 28263 | Anemia | NULL | NULL |
| 245 | 28264 | Anemia | NULL | NULL |
| 246 | 28268 | Anemia | NULL | NULL |
| 247 | 28269 | Anemia | NULL | NULL |
| 248 | 2827 | Anemia | NULL | NULL |
| 249 | 2828 | Anemia | NULL | NULL |
| 250 | 2829 | Anemia | NULL | NULL |
| 251 | 283 | Anemia | NULL | NULL |
| 252 | 2830 | Anemia | NULL | NULL |
| 253 | 2831 | Anemia | NULL | NULL |
| 254 | 28310 | Anemia | NULL | NULL |
| 255 | 28311 | Anemia | NULL | NULL |
| 256 | 28319 | Anemia | NULL | NULL |
| 257 | 2832 | Anemia | NULL | NULL |
| 258 | 2839 | Anemia | NULL | NULL |
| 259 | 284 | Anemia | NULL | NULL |
| 260 | 2840 | Anemia | NULL | NULL |
| 261 | 28401 | Anemia | NULL | NULL |
| 262 | 28409 | Anemia | NULL | NULL |
| 263 | 2841 | Anemia | NULL | NULL |
| 264 | 2842 | Anemia | NULL | NULL |
| 265 | 2848 | Anemia | NULL | NULL |
| 266 | 28481 | Anemia | NULL | NULL |
| 267 | 28489 | Anemia | NULL | NULL |
| 268 | 2849 | Anemia | NULL | NULL |
| 269 | 285 | Anemia | NULL | NULL |
| 270 | 2850 | Anemia | NULL | NULL |
| 271 | 2851 | Anemia | NULL | NULL |
| 272 | 2852 | Anemia | NULL | NULL |
| 273 | 28521 | Anemia | NULL | NULL |
| 274 | 28522 | Anemia | NULL | NULL |
| 275 | 28529 | Anemia | NULL | NULL |
| 276 | 2853 | Anemia | NULL | NULL |
| 277 | 2858 | Anemia | NULL | NULL |
| 278 | 2859 | Anemia | NULL | NULL |
| 279 | 491 | COPD | NULL | NULL |
| 280 | 492 | COPD | NULL | NULL |
| 281 | 493 | COPD | NULL | NULL |
| 282 | 496 | COPD | NULL | NULL |
| 283 | 4910 | COPD | NULL | NULL |
| 284 | 4911 | COPD | NULL | NULL |
| 285 | 4912 | COPD | NULL | NULL |
| 286 | 49120 | COPD | NULL | NULL |
| 287 | 49122 | COPD | NULL | NULL |
| 288 | 4918 | COPD | NULL | NULL |
| 289 | 4919 | COPD | NULL | NULL |
| 290 | 4920 | COPD | NULL | NULL |
| 291 | 4928 | COPD | NULL | NULL |
| 292 | 4930 | COPD | NULL | NULL |
| 293 | 49300 | COPD | NULL | NULL |
| 294 | 49301 | COPD | NULL | NULL |
| 295 | 49302 | COPD | NULL | NULL |
| 296 | 4931 | COPD | NULL | NULL |
| 297 | 49311 | COPD | NULL | NULL |
| 298 | 49312 | COPD | NULL | NULL |
| 299 | 4932 | COPD | NULL | NULL |
| 300 | 49320 | COPD | NULL | NULL |
| 301 | 49321 | COPD | NULL | NULL |
| 302 | 49322 | COPD | NULL | NULL |
| 303 | 4938 | COPD | NULL | NULL |
| 304 | 49381 | COPD | NULL | NULL |
| 305 | 49382 | COPD | NULL | NULL |
| 306 | 4939 | COPD | NULL | NULL |
| 307 | 49390 | COPD | NULL | NULL |
| 308 | 49391 | COPD | NULL | NULL |
| 309 | 49392 | COPD | NULL | NULL |
| 310 | 584 | Acute renal failure | NULL | NULL |
| 311 | 5845 | Acute renal failure | NULL | NULL |
| 312 | 5846 | Acute renal failure | NULL | NULL |
| 313 | 5847 | Acute renal failure | NULL | NULL |
| 314 | 5848 | Acute renal failure | NULL | NULL |
| 315 | 5849 | Acute renal failure | NULL | NULL |
| 316 | 5851 | CKD | NULL | NULL |
| 317 | 5852 | CKD | NULL | NULL |
| 318 | 5853 | CKD | NULL | NULL |
| 319 | 5854 | CKD | NULL | NULL |
| 320 | 5855 | CKD | NULL | NULL |
| 321 | 5856 | CKD | NULL | NULL |
| 322 | 5859 | CKD | NULL | NULL |
| 323 | 44389 | PVD | NULL | NULL |
| 324 | 4409 | PVD | NULL | NULL |
| 325 | 4439 | PVD | NULL | NULL |
| 326 | 4442 | PVD | NULL | NULL |
| 327 | 44422 | PVD | NULL | NULL |
| 328 | 4448 | PVD | NULL | NULL |
| 329 | 44502 | PVD | NULL | NULL |
| 330 | 4471 | PVD | NULL | NULL |
| 331 | 5570 | PVD | NULL | NULL |
| 332 | 5571 | PVD | NULL | NULL |
| 333 | 4402 | PVD | NULL | NULL |
| 334 | 4403 | PVD | NULL | NULL |
| 335 | 2507 | PVD | NULL | NULL |
| 336 | 4403 | PVD | NULL | NULL |
| 337 | 7071 | PVD | NULL | NULL |
| 338 | 44021 | PVD | NULL | NULL |
| 339 | 44022 | PVD | NULL | NULL |
| 340 | 44023 | PVD | NULL | NULL |
| 341 | 44024 | PVD | NULL | NULL |
| 342 | 44029 | PVD | NULL | NULL |
| 343 | 4404 | PVD | NULL | NULL |
| 344 | 4408 | PVD | NULL | NULL |
| 345 | 44030 | PVD | NULL | NULL |
| 346 | 44031 | PVD | NULL | NULL |
| 347 | 44032 | PVD | NULL | NULL |
| 348 | 25070 | PVD | NULL | NULL |
| 349 | 25071 | PVD | NULL | NULL |
| 350 | 25072 | PVD | NULL | NULL |
| 351 | 25073 | PVD | NULL | NULL |
| 352 | 4450 | PVD | NULL | NULL |
| 353 | 44501 | PVD | NULL | NULL |
| 354 | 44502 | PVD | NULL | NULL |
| 355 | 4458 | PVD | NULL | NULL |
| 356 | 44581 | PVD | NULL | NULL |
| 357 | 44589 | PVD | NULL | NULL |
| 358 | 4414 | PVD | NULL | NULL |
| 359 | 4400 | PVD | NULL | NULL |
| 360 | 4440 | PVD | NULL | NULL |
| 361 | 3042 | Cocaine/amphetamine | NULL | NULL |
| 362 | 30420 | Cocaine/amphetamine | NULL | NULL |
| 363 | 30421 | Cocaine/amphetamine | NULL | NULL |
| 364 | 30422 | Cocaine/amphetamine | NULL | NULL |
| 365 | 30423 | Cocaine/amphetamine | NULL | NULL |
| 366 | 3044 | Cocaine/amphetamine | NULL | NULL |
| 367 | 30440 | Cocaine/amphetamine | NULL | NULL |
| 368 | 30441 | Cocaine/amphetamine | NULL | NULL |
| 369 | 30442 | Cocaine/amphetamine | NULL | NULL |
| 370 | 30443 | Cocaine/amphetamine | NULL | NULL |
| 371 | 3040 | Opioid | NULL | NULL |
| 372 | 30400 | Opioid | NULL | NULL |
| 373 | 30401 | Opioid | NULL | NULL |
| 374 | 30402 | Opioid | NULL | NULL |
| 375 | 30403 | Opioid | NULL | NULL |
| 376 | 3030 | Alcohol abuse | NULL | NULL |
| 377 | 30300 | Alcohol abuse | NULL | NULL |
| 378 | 30301 | Alcohol abuse | NULL | NULL |
| 379 | 30302 | Alcohol abuse | NULL | NULL |
| 380 | 30303 | Alcohol abuse | NULL | NULL |
| 381 | 3039 | Alcohol abuse | NULL | NULL |
| 382 | 30390 | Alcohol abuse | NULL | NULL |
| 383 | 30391 | Alcohol abuse | NULL | NULL |
| 384 | 30392 | Alcohol abuse | NULL | NULL |
| 385 | 30393 | Alcohol abuse | NULL | NULL |
| 386 | 3050 | Alcohol abuse | NULL | NULL |
| 387 | 30500 | Alcohol abuse | NULL | NULL |
| 388 | 30501 | Alcohol abuse | NULL | NULL |
| 389 | 30502 | Alcohol abuse | NULL | NULL |
| 390 | 30503 | Alcohol abuse | NULL | NULL |
| 391 | 3055 | Opioid | NULL | NULL |
| 392 | 30550 | Opioid | NULL | NULL |
| 393 | 30551 | Opioid | NULL | NULL |
| 394 | 30552 | Opioid | NULL | NULL |
| 395 | 30553 | Opioid | NULL | NULL |
| 396 | 3056 | Cocaine/amphetamine | NULL | NULL |
| 397 | 30561 | Cocaine/amphetamine | NULL | NULL |
| 398 | 30562 | Cocaine/amphetamine | NULL | NULL |
| 399 | 30563 | Cocaine/amphetamine | NULL | NULL |
| 400 | 3057 | Cocaine/amphetamine | NULL | NULL |
| 401 | 30570 | Cocaine/amphetamine | NULL | NULL |
| 402 | 30571 | Cocaine/amphetamine | NULL | NULL |
| 403 | 30572 | Cocaine/amphetamine | NULL | NULL |
| 404 | 30573 | Cocaine/amphetamine | NULL | NULL |
| 529 | 42731 | AF | NULL | NULL |
| 530 | 4271 | VT | NULL | NULL |
| 531 | 4275 | Cardiac arrest | NULL | NULL |
| 532 | 412 | CAD | NULL | NULL |

Abbreviations: CAD, Coronary artery disease; COPD, chronic obstructive pulmonary disease; CKD, chronic kidney disease; PVD, peripheral vascular disease; AF, atrial fibrillation; VT, ventricular tachycardia.
