# Supplementary material for: Marijuana use and short-term outcomes in patients hospitalized for acute myocardial infarction
Source: PLoS One. 2018 Jul 11;13(7):e0199705. doi: 10.1371/journal.pone.0199705 (PMC6040751; doi:10.1371/journal.pone.0199705)
Supplement: S3 Table — (DOCX) [file pone.0199705.s003.docx]

**S3 Table. Patient Characteristics of the Original Study Population**

| N=3097294 | Marijuana,  N (%)*  8673 (0.28) | No marijuana,  N (%)  3088621 (99.72) | p-value |
| --- | --- | --- | --- |
| Age group‡  <18  18-29  30-39  40-49  50-59  60-69  >70  NA | 51 (1)  422 (5)  998 (12)  2065 (24)  2079 (24)  777(9)  172(2)  2109 (24) | 1706 (0)  7265 (0)  46534 (2)  218107 (7)  455972 (15)  588388 (19)  1568027 (51)  202622 (7) | <0.001 |
| Race‡  White  AA  Asian  Other  Unknown  Hispanic  NA | 3859 (45)  1784 (21)  35 (0)  272 (3)  169 (2)  875 (10)  1679 (19) | 1928518 (62)  219805 (7)  71172 (2)  10709 (4)  86664 (3)  281289 (9)  393364(13) | <0.001 |
| Payer‡  Medicare  Medicaid  Private  Workers Comp  County/Indigent  Other Government  Self pay  Other  NA | 1369 (16)  1851 (21)  2737 (32)  16 (0)  465 (5)  167 (2)  1646 (19)  220 (3)  202 (2) | 1877755 (61)  191882 (6)  791042 (26)  5018 (0)  22090 (1)  20950 (1)  114739 (4)  45936 (2)  19209 (1) | <0.001 |
| Comorbidities  Anemia‡  Chronic kidney disease‡  Hypertension‡  Hyperlipidemia‡  Heart failure‡  COPD*  Coronary artery disease  Atrial fibrillation‡  Diabetes Mellitus‡  Alcohol‡  Tobacco‡  Cocaine/methamphetamine‡ | 1092 (13)  498 (6)  4490 (52)  3292 (38)  1501 (17)  1353 (16)  5117 (59)  451 (5)  1575 (18)  1946 (22)  5266 (61)  1886 (22) | 607807 (20)  216093 (7)  1832373 (59)  1050397 (34)  1096528 (36)  509771 (17)  1807765 (59)  557058 (18)  946802 (31)  66231 (2)  596911 (19)  13280 (0) | <0.001  <0.001  <0.001  <0.001  <0.001  0.023  0.376  <0.001  <0.001  <0.001  <0.001  <0.001 |

*Data are presented N (%) unless otherwise indicated.

Abbreviations: AA, African Ancestry; COPD, Chronic obstructive pulmonary disease; *p<=0.05 †p<0.01 ‡p<0.001
