# Supplementary material for: Marijuana use and short-term outcomes in patients hospitalized for acute myocardial infarction
Source: PLoS One. 2018 Jul 11;13(7):e0199705. doi: 10.1371/journal.pone.0199705 (PMC6040751; doi:10.1371/journal.pone.0199705)
Supplement: S1 Fig — (DOCX) [file pone.0199705.s004.docx]

**S1 Fig: Multivariate Odds Ratios of Outcomes in Patients with Concurrent Cardiotoxic Substance Use and in Patients > 70 Years of Age.**

**
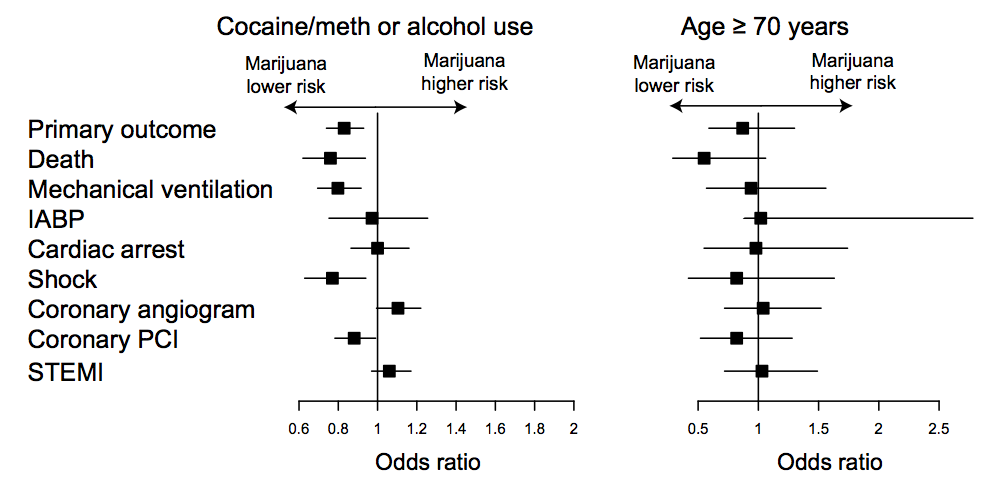
**

Abbreviations: Vent, Mechanical ventilation; IABP, Intraaortic balloon pump; VF, Ventricular fibrillation; VT, Ventricular tachycardia; Coronary angio, Coronary angiogram; PCI, Percutaneous coronary intervention; STEMI= ST elevation myocardial infarction
