# Supplementary material for: Marijuana use and short-term outcomes in patients hospitalized for acute myocardial infarction
Source: PLoS One. 2018 Jul 11;13(7):e0199705. doi: 10.1371/journal.pone.0199705 (PMC6040751; doi:10.1371/journal.pone.0199705)
Supplement: S2 Fig — Primary outcome includes: in-hospital death, intraaortic balloon pump placement, (IABP), mechanical ventilation, cardiac arrest, and shock. (DOCX) [file pone.0199705.s005.docx]

**
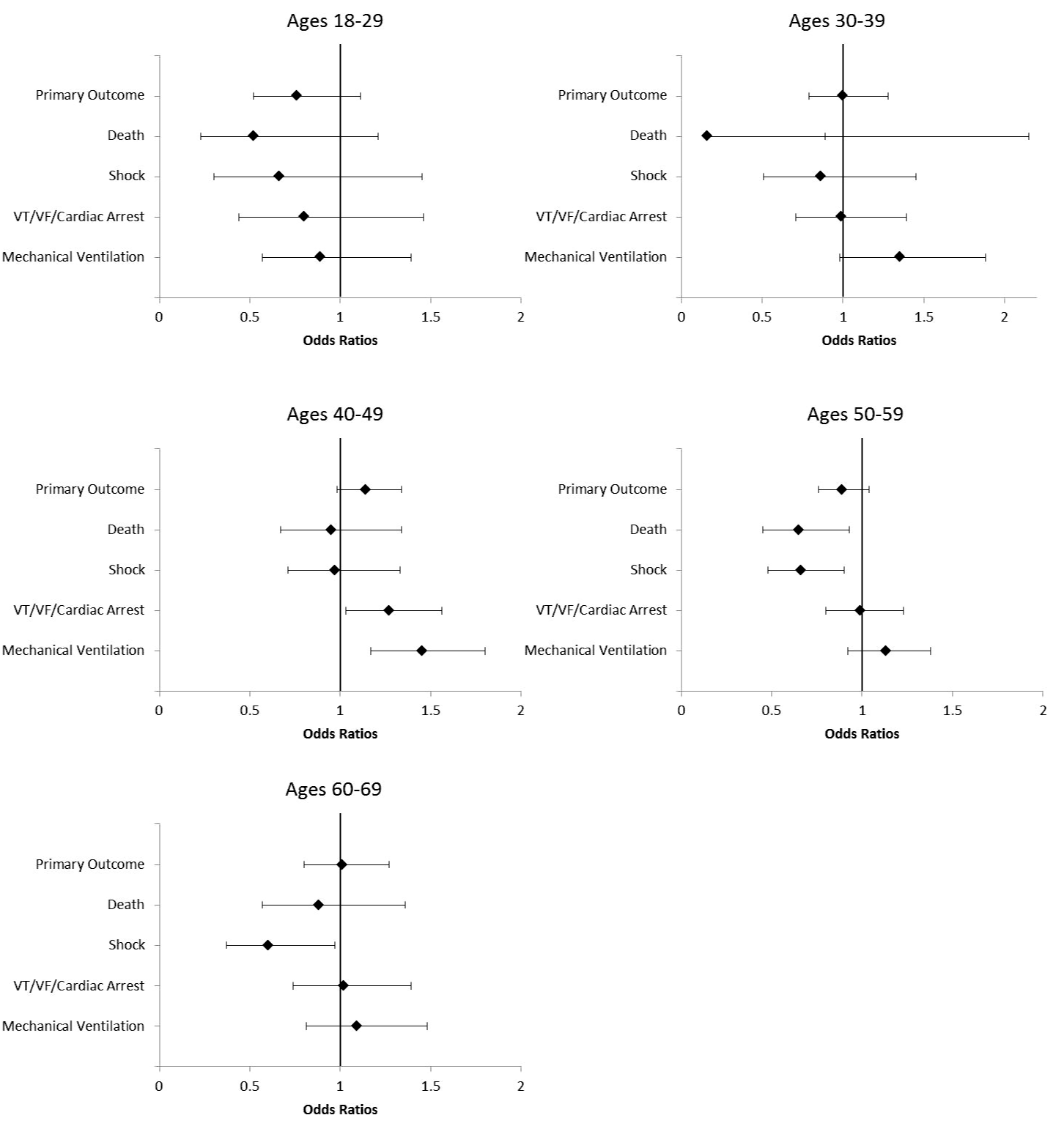
**

**S2 Fig. Age-Specific analysis of outcomes.** Primary outcome includes: in-hospital death, intraaortic balloon pump placement, (IABP), mechanical ventilation, cardiac arrest, and shock.
